# Rett Syndrome: A Tale of Altered Genetics, Synaptic Plasticity, and Neurodevelopmental Dynamics

**DOI:** 10.7759/cureus.41555

**Published:** 2023-07-08

**Authors:** David C Oluigbo

**Affiliations:** 1 Department of Brain and Cognitive Sciences, Massachusetts Institute of Technology, Cambridge, USA

**Keywords:** altered neurodevelopment, synaptic plasticity, mecp2-null mice models, mecp2 mutation, rett syndrome

## Abstract

Rett syndrome (RTT) is a neurodevelopmental disorder that is a leading cause of severe cognitive and physical impairment. RTT typically occurs in females, although rare cases of males with the disease exist. Its genetic cause, symptoms, and clinical progression timeline have also become well-documented since its initial discovery. However, a relatively late diagnosis and lack of an available cure signify that our understanding of the disease is incomplete. Innovative research methods and tools are thereby helping to fill gaps in our knowledge of RTT. Specifically, mouse models of RTT, video analysis, and retrospective parental analysis are well-established tools that provide valuable insights into RTT. Moreover, current and anticipated treatment options are improving the quality of life of the RTT patient population. Collectively, these developments are creating optimistic future perspectives for RTT.

## Introduction and background

Rett syndrome (RTT) is a progressive neurodevelopmental disorder that is the leading cause of severe intellectual disability in females and has an incidence of approximately 1 in 10,000 females. The disease can also occur in males, but most males with RTT die before birth or during infancy. The majority of the RTT patient population is female as a result. RTT is characterized by a brief period of apparently normal development before stagnation. Neurodevelopmental regression is accompanied by a loss of previously acquired skills, such as motor control and speech, along with the manifestation of neurological symptoms like anxiety and seizures [1].

Dr. Andreas Rett first described RTT in 1966, and the disease has since garnered significant attention in scientific research. Dr. Bengt Hagberg’s 1983 report based on his investigation of 35 patients marked the first published report of RTT. Dr. Huda Zoghbi and his collaborators then identified mutations of the methyl CpG binding protein 2 (MECP2) gene in RTT patients in 1999 [2]. MECP2 is an X-linked gene that encodes MeCP2, a DNA methylation reader protein with functions that include transcriptional repression, transcriptional activation, and RNA binding. MECP2 mutations account for approximately 95% of typical RTT cases, and approximately, 600 RTT-linked MECP2 mutations have been identified thus far [3]. These identified MECP2 mutations include missense and nonsense mutations, insertions, and deletions. The impact of these MECP2 mutations on the severity of RTT phenotypes varies depending on X-linked gene deactivation, potentially leading to more severe manifestations of RTT. Mutated MECP2 encodes dysfunctional MeCP2 that is limited in its ability to act as effective transcriptional and post-transcriptional regulators. RTT-linked MECP2 mutations also disrupt brain development during neurogenesis and chromatin modification related to MeCP2’s C-terminal domain (CTD) [4]. Moreover, MECP2 mutations undermine synaptic connections, experience-dependent synaptic plasticity, and maintenance of neural function in adulthood [5-10]. Mutations in other genes can also produce RTT or RTT-like phenotypes. Forkhead box G1 (FOXG1) and cyclin-dependent kinase-like 5 (CDKL5) gene mutations have been observed in atypical RTT variants in particular [11]. However, MECP2 will be the primary gene discussed because most RTT cases exhibit a mutation in this gene.

In this paper, we review the progress in our understanding of the etiology and clinical progression of RTT. We consider the influence of RTT-linked mutations on the development of synaptic connections, synaptic plasticity, and consequent neurodevelopmental dynamics. We then shift our discussion to the roles of RTT animal models, video analysis, and retrospective parental analysis in enhancing our understanding of RTT. Finally, we investigate developments in treatment and diagnosis practices that have emerged alongside our increased knowledge.

## Review

Etiology

RTT largely stems from the mutation of the MECP2 gene, which encodes a protein called MeCP2. MeCP2 binds methylated DNA and is expressed in different tissues throughout the body. It is most highly concentrated in the brain and neurons of mammals though [10]. For example, adult male mice have more than 16 million MeCP2 molecules in neuron nuclei compared to approximately 500,000 MeCP2 molecules in their liver hepatocyte nuclei [12]. In addition to its widespread distribution throughout the body, MeCP2 is similarly diverse in its physiological roles. MeCP2 acts as a transcriptional regulator, although its exact targets and interactions are still unclear. It is known for its binding to methylated DNA at GG and CA dinucleotides throughout the genome [12-16]. Hence, researchers have suggested that MeCP2 may act as a transcriptional repressor by recruiting transcriptional co-repressors such as nuclear receptor corepressor 1 (NCOR1) to specific methylated DNA sites [12,17-19]. Recent studies have also proposed that MeCP2 preferentially binds to and represses transcription of long genes (100 kb or greater) that are highly methylated due to their higher levels of methylated CA (mCA) gene bodies [13,15,16,20]. MeCP2 may be further involved in transcriptional regulation as a transcriptional activator due to its proposed interaction with cAMP response element binding protein (CREB), a transcriptional activator [21]. Besides transcriptional regulation, MeCP2 can act as a post-transcriptional regulator due to its post-transcriptional splicing modulation and suppression of nuclear microRNA processing. The protein can also act as a regulator in synaptic activity and development, a binding partner of 5-methylcytosine (5mC), and 5hmC binding protein within the brain [21-26]. MeCP2’s ability to carry out this diverse array of physiological functions is disrupted by loss of function MECP2 mutations, however. Such mutations undermine the gene’s ability to produce functional MeCP2 and cause individuals to develop the characteristic Rett syndrome symptoms discussed later.

From the nearly 600 identified MECP2 RTT-linked mutations, eight missense, and nonsense mutations, R106W, R133C, T158M, R168X, R255X, R270X, R294X, and R306C account for more than 60% of RTT disease cases [3]. The reason for the preponderance of these mutations in RTT disease cases is still unclear. Most MECP2 mutations are de-novo and spontaneously occur in the paternal germ line, meaning that RTT is typically not a heritable disorder [26]. Since MECP2 is an X-linked gene, its loss of function mutation proves lethal in most males before birth. Of the small proportion of males who complete prenatal development, the majority die from congenital encephalopathies linked to loss-of-function MECP2 mutation [27]. However, there are case reports of males who have survived RTT beyond prenatal and early postnatal development [28-32]. In contrast, approximately 1 in 10,000 live female births result in RTT-linked loss-of-function MECP2 mutations [33]. Affected females have somatic cells that contain one inactivated X chromosome due to X-chromosome inactivation. Female RTT patients thereby exhibit mosaic expression of MECP2 mutations because only a subset of their cells expresses mutated MECP2 genes. X-chromosome inactivation is only one of the contributors to clinical variability observed in female RTT patients [26]. Other possible contributors include the type of mutation involved and epigenetic mechanisms [34-36]. Besides X-chromosome inactivation, another feature distinguishing female RTT patients from their male counterparts is their expected lifespan. Many females with RTT live into adulthood despite physical and mental issues related to stagnated development and regression. Many prevalence or incidence statistics thereby consider only females because they represent a disproportionate amount of RTT cases.

MECP2 mutations are not the only genetic mutations observed in RTT patients. Patients diagnosed with atypical RTT may exhibit CDKL5 mutations. CDKL5 is an X-linked gene that encodes a serine/threonine kinase mainly expressed in the brain, testes, and thymus [37]. This kinase moves between different brain regions during development, has peak expression during the early postnatal period, and is largely expressed within neuronal dendrites and nuclei. Its long C-terminal tail may regulate this pattern of distribution [38]. The kinase also plays a role in neuronal migration, cell proliferation, synaptic development, and axon outgrowth [39]. These processes are disrupted in the case of CDKL5 mutations which share parallels with MECP2 mutations. CDKL5 mutations are connected to core RTT symptoms, including developmental delay, seizures, speech problems, and limited motor function. They also are not invariably lethal for males even though they are hemizygous for the X-linked gene [40]. Case reports of male pediatric patients with CDKL5 mutations exist [38,41]. CDKL5 and MECP2 mutations also differ in several ways. Spinal curvature and regression are less commonly associated with CDKL5 mutations than MECP2 mutations [42,43]. Moreover, CDKL5 mutations are associated with the Hanefeld variant of RTT known for its hallmark of early-onset seizures as opposed to typical RT [44].

Besides CDKL5 mutations, patients diagnosed with atypical RTT may carry FOXG1 mutations. The FOXG1 gene is located on chromosome 14q12 and encodes a forkhead box protein G1 (FoxG1). FoxG1 is a transcription factor that is not only integral to the development of the ventral telencephalon but also to the regulation of neuronal proliferation and differentiation [45-47]. FOXG1 mutations include deletions, frameshift, duplications, and point mutations, and more than 120 different mutations have been identified [48-51]. These mutations can result in different clinical phenotypes similar to MECP2 mutations. For instance, clinical manifestations of epilepsy and movement disorders can differ between patients with FOXG1 duplications versus deletions [48,52]. However, RTT-linked FOXG1 mutations are associated with the congenital variant of RTT, a condition characterized by earlier disease onset and development delay compared to typical RTT cases [53]. In addition to understanding these genetic variations associated with the etiology of RTT, it is important to consider the clinical progression of Rett syndrome.

Clinical presentation

The clinical progression of RTT typically consists of four progressive stages. These four stages are stagnation (6-18 months of age), rapid regression (1-4 years of age), pseudo-stationary period (2 years of age potentially life), and motor deterioration (10 years of age-life). Furthermore, the severity of symptoms during this progression timeline depends on multiple factors, including the degree of X-linked inactivation in each RTT patient, epigenetic factors, and the type of mutation involved. RTT patients first experience normal development for about the first six months of infancy, although there may be subtle demonstrations of RTT symptoms. Subtle demonstrations of RTT symptoms may include hypotonia or lack of interest in other people even before this six-month benchmark [54]. Most RTT individuals then experience stagnation in their neurodevelopment between 6 and 18 months of age. This period marks the appearance of more noticeable RTT symptoms. These symptoms include but are not limited to less eye contact, more frequent hand wringing, reduced head growth, and delayed motor development [55]. RTT patients then experience rapid regression between one and four years of age, marked by loss of previously acquired skills and development of new symptoms. Stereotypical hand motions may replace purposeful ones and include more frequent wringing, rubbing, and scratching [54,56]. Loss of the ability to walk can confine patients to wheelchair use. Respiratory issues, including hyperventilation and breath-holding episodes, may develop. Varying severities of anxiety may manifest depending on an individual’s mutation [57]. Approximately 60% of RTT individuals may also develop seizures, typically beginning at 2-3 years of age or older [58,59]. Risk factors for epilepsy in RTT patients include the development of microcephaly during infancy and lack of the ability to walk [60,61]. Patients most frequently exhibit generalized tonic-clonic, tonic, myoclonic, and complex partial seizures [62]. Less common seizure types in RTT cases include absence and clonic seizures [60,63]. Additionally, RTT patients can exhibit focal epilepsy or generalized epilepsy, but focal epilepsy appears to be more common among them [64].

RTT patients eventually reach a pseudo-stationary stage where regression largely stops [55]. The primary exception is motor regression which may proceed at a slower rate. Many patients cannot live independently at this stage due to physical and cognitive impairment. They often experience behavioral issues, impaired motor coordination, limited mobility, stereotypical hand motions, and breathing irregularities linked to earlier regression [65,66]. Possible clinical features include but are not limited to scoliosis, shortening of the Achilles tendon, ankle deformation, sudden agitation, sleep cycle disturbances, and nighttime laughing. Besides physical and cognitive limitations, patients can also improve in specific RTT symptoms. Patients who developed epilepsy during the rapid regression may become seizure-free. Visual contact behavior may improve, allowing individuals to communicate more effectively with eye-pointing behavior. Social awareness might even improve as a patient’s alertness increases. Although pseudo-stationary is the final developmental stage for some RTT patients, others experience one more progressive stage. This last stage is called late motor deterioration and can last years to decades. If not already confined to a wheelchair, patients might come to rely on one due to severe physical impairment from muscle weakness, spasticity, and rigidity [55]. Eye pointing and visual contact behaviors persist despite that, so patients are still capable of communication.

Pathophysiological insights from mouse models of MECP2 mutation

MECP2-mutant mice lines are currently the primary animal model used to study RTT. The MECP2 gene is only present in vertebrates like mice, making non-vertebrae models like fruit flies impractical [56]. Mecp2tm1.1Bird and Mecp2tm1.1Jae are the two most common mice lines used in MECP2-mutant models. MECP2 is available in very small amounts in Mecp2tm1.1Jae mice, whereas it is absent from Mecp2tm1.1Bird mice [65,67]. This is likely due to the deletions of exons 3 and 4 in Mecp2tm1.1Bird and the deletion of exon 3 in Mecp2tm1.1Jae [65,67]. Both cases may seem too extreme to represent RTT and its symptoms. For reference, most MECP2 mutations (about 70%) involve one or more of the eight most common RTT-linked nonsense or missense mutations, and very few produce the complete deletion of the MECP2 gene [3]. These models, nevertheless, have proved accurate recreations of RTT’s associated phenotype.

Since most RTT patients are females, female Mecp2 mice with mutated MECP2 closely resemble the symptoms and clinical progression of RTT. However, one factor makes it much more difficult for researchers to study the phenotypes of these female mice: X-chromosome inactivation (XCI) skewing [55]. Absent from male mouse models, XCI skewing results in X-chromosome inactivation that is heavily weighted toward one chromosome, usually the maternal chromosome. This wider range of phenotype in the female mice presents challenges in terms of analyzing MECP2 mutant mouse models. However, it also grants researchers the opportunity to study varied clinical phenotypes due to the heterogeneous nature of the underlying XCI skewing. In contrast, hemizygous Mecp2-mutant male mouse models have less variation in underlying genetic determinants, allowing a more mechanistic study of a smaller set of RTT phenotypes.

Like human infants with RTT, hemizygous Mecp2-mutant male mice initially experience normal development. As opposed to the six months of human babies, their period of normal development is approximately the first four weeks of their life [65,67]. Following these four weeks, male mice rapidly regress until they die around 6-12 months of age [55]. MECP2-mutant male mice develop RTT-like symptoms during this regression phase, including breathing issues, hind limb clasping, decreased motor coordination, seizures, reduced brain volume, and trembling [65,67]. These clear parallels show that hemizygous Mecp2-mutant male mice still capture symptoms associated with RTT despite their hemizygosity and lack of XCI skewing. Heterozygous MeCP2 mutant female mice experience a similar developmental trajectory at a delayed age of 4 - 6 months, and they have a normal lifespan [65,67]. The observed phenotypes of abnormal neurodevelopment in their cognitive and motor domains are due to underlying changes in postnatal synaptic plasticity and brain circuitry influenced by abnormal regulation by mutated MECP2. These properties of MECP2 mutant mouse models have led to their integration into numerous investigations. 

Neurophysiology: impact on postnatal synaptic plasticity, brain circuitry, and neurodevelopment

MeCP2 deficiency in the case of RTT-linked MECP2 mutations is linked to altered neurophysiology. One consequence of decreased MeCP2 expression is morphological changes in the cerebral cortex observed in humans and mice. Shorter dendrite lengths have been noted in the pyramidal neurons in the motor and frontal cortices of human RTT individuals compared to the brains of non-RTT individuals [28]. Reduced volume in the parietal and temporal lobes of female RTT patients compared to their non-RTT counterparts has also been identified via MRI scanning [68]. Altered cortical development has even been identified in postmortem analysis of RTT patients. Specifically, cortical areas of RTT brains analyzed postmortem exhibited fewer dendritic spines. MECP2 mutant mouse models similarly demonstrate alterations in cortical development. Eight-week-old MeCp2 -/Y mice exhibited decreased thickness and increased cell density within their neocortical layers. This outcome suggests that MeCP2 deficiency instead of corticogenesis deficits may be the underlying cause due to MeCP2’s expression during early neurogenesis. Besides morphological changes, MECP2 mutations can delay corticogenesis processes. Examples of processes that may be delayed are migration from subventricular and ventricular regions to the cortical plate [69].

In addition to cortical development, RTT undermines proper synaptic plasticity. To understand RTT’s impact on these synaptic properties, researchers have largely used two mouse models: mice with global MECP2 deletion and mice with MECP2 deleted from specific cells and neuronal types such as astrocytes [70]. Post-tetanic potentiation (PTP) and paired-pulse facilitation are forms of short-term plasticity that are disrupted in MECP2-deficient mouse models upon disease onset [71]. Long-term depression (LTD) and long-term potentiation (LTP) may also be impaired in the case of MECP2 mutations. Hippocampal CA3-CA1 synapses of MECP2 knockout (KO) mice exhibit dysfunctional LTD and LTP. LTP is also disrupted in the motor cortex of RTT individuals who experience hyperexcitation related to dysfunctional GABAergic activity [72]. In the hippocampal CA2, LTP has also been impaired in CA3 pyramidal neurons of MECP2-KO mice from the Bird line because of an altered GABAergic developmental shift [73]. Besides short- and long-term plasticity, homeostatic synaptic plasticity may be adversely impacted by MeCP2 deficiency. Synaptic scaling up and down, two forms of homeostatic plasticity, are connected to MeCP2 upregulation and can be impaired in the case of MeCP2 deficiency. One study found that hippocampal neurons from MECP2 KO mice exhibited impaired synaptic scaling down during bicuculline treatment [25]. Close monitoring of GluA1 trafficking in MECP2 KO neurons treated with TTX and bicuculine similarly reveals impaired synaptic scaling up and down.

Another consequence of RTT-linked MECP2 mutations is its impact on excitation/inhibition (E/I). Increased excitation can influence network oscillations and increase susceptibility to seizures [58,64,74]. Several studies also suggest that levels of several brain circuitry components may also be offered. Decreased inhibition has been linked to lower amounts of miniature inhibitory postsynaptic currents (mIPSCs) in Cornu Ammonis (CA) pyramidal neurons in MECP2 models with global deletion [21,75-77]. An increase in perisomatic GABAergic terminals and a decrease in excitatory projections have also been identified in the cortex and hippocampus. Moreover, both decreases and increases in miniature excitatory postsynaptic currents (mEPSCs) in the brain [6,75,78,79]. The visual cortex and brainstem are no exception to this pattern of altered circuitry. Brainstems of RTT patients can express hyperexcitable neurons and reduced GABAergic inhibition, which is the root of breathing issues [80]. In the visual cortex, MeCP2 deficiency impacts the transcription of GluN2B, a subunit of N-methyl-D-aspartic acid receptors (NMDARs) replaced with another subunit called GluN2A [81]. MeCP2 mediates the inhibition of GluN2B's transcription, meaning MeCP2 deficiency is associated with an altered growth rate of the GluN2A/Glun2B ratio. An accelerated growth rate of the GluN2A/GluN2B ratio in the parvalbumin (PV) + interneurons of a MeCP2 deficient visual cortex has been associated with vision regression [82,83]. It is important to note that studies investigating the neurophysiology of RTT are not the only tools advancing our understanding of the disease.

Clinical studies

Insights Into Clinical Neurodevelopmental Trajectory in RTT From Video Analysis

In addition to neurodevelopmental analysis, family-acquired videos have become another method of studying the behavior and regression of RTT patients. Recorded when most parents or guardians are not fully aware of the child’s neurodevelopment disorder, family footage provides an in-depth look into behavioral issues through the objective lens of a camera. Since the 1990s, researchers have gradually learned to manipulate video-audio recordings to their advantage in different experimental setups. For example, one 2005 study involved Heinz Prechtl and Christa Einspieler analyzing 17 family videos of infants during their first six months of life [84]. Of the 17 infants recorded, 8 were females later diagnosed with RTT while the remaining 9 were "apparently" normally developing children [84]. For their experiment, the development outcome of each infant was withheld from Einspieler and Prechtl [84]. Still, both researchers accurately determined which babies had RTT versus which ones were "apparently" normally developing through early motor and communication behavior [84]. Family footage is not only a means of using behavioral cues to distinguish RTT individuals from their "apparently" normally developing peers.

Recordings have also paved the way to understanding more about the clinical characteristics of RTT. Video analysis has increased understanding of the speech abnormalities associated with RTT. Specifically, analysis of footage of three-year-old toddlers with RTT’s preserved speech variant (PSV) shows how abnormal vocalization patterns can interrupt normal babbling [85-87]. Recordings also provide valuable information for observing impaired motor abilities that are common symptoms of RTT. Clips of 9-12-month-old infants with RTT have shown pincer grasping occasionally interrupted by stereotypical hand motions that only become more frequent as children grew older [85,88]. Finally, video data can be cross-referenced with additional information about subjects to draw insightful conclusions about certain clinical features. Researchers have employed this strategy to investigate the relationship between phenotypes and different RTT-linked mutations over different ages. In one video data study featuring video data from 99 female individuals, Dr. Downs and her colleagues used genetic testing results to obtain the distribution of MECP2 mutations among subjects [89]. Her group then used video recordings to score the motor profile of each subject, and they paired these scores with each subject’s genotype. Their findings demonstrate specific MECP2 mutations corresponding to more severe impairment of complex motor skills in subjects younger than 13 years of age and 13 years of age and older [89]. Furthermore, the researchers were able to use the subjects’ ages to see if the mutations associated with better general mobility and complex motor skills were the same between their two age groups. 

Family video analysis still has its limitations. Foremost, videos cannot consistently produce comparable data due to a lack of standardized conditions. This can be improved by standardizing the conditions and way the videos are obtained. Parents do not normally care which environment they are filming in as long they can preserve memorable moments or milestones for later recollection. Since these videos lack a standardized setting, researchers can find it difficult to compare behavioral observations between different videos [90-92]. Secondly, many home videos were not taken with the expectation or purpose of later scientific analysis since most parents and caregivers were unaware of their child’s condition. Footage quality and quantity can be inconsistent at best, hindering a researcher’s ability to determine the age of a child with precision [93]. Finally, cameras are only an effective source of information in this context when they are recording. With family clips ranging from seconds to minutes long, the camera does not capture the full range of a child’s behavior. Some behavioral patterns that mark other deviations from normal development in RTT patients might escape the film. The impact of the limitations of family video analysis and the extensive amount of video sampling required to make specific assessments on neurodevelopmental trajectories remains unclear and is the subject of ongoing research.

Insights Into Neurodevelopmental Dynamics and Trajectory From Retrospective Parent Analysis

Parental interviews and inquiries are additional established methods of studying RTT. Both forms of retrospective parental analysis incorporate a typical question-and-answer format. Responses of parents are recorded and referenced in later statistical analysis. The main aim of these procedures is to shed more light on the early development of RTT patients by asking individuals who can provide detailed accounts of this period. Parents can provide their accounts of atypical behaviors that their children might have acquired during “normal” development, stagnation, or regression phases of RTT. Unlike a recorded video clip, parents can also highlight frequently occurring behavior abnormalities not captured by a video camera. For these reasons, parental interviews and inquiries have been useful in advancing the understanding of RTT progression in the recent past. In a retrospective analysis inquiry performed by Dr. Kerr, nearly half of the parents noted that they noticed something unusual about their child’s development before six months of age [94]. Common comments by parents include their children being excessively calm, needing to be woken up to feed, having an empty gaze, giving feeding problems, and attaining developmental milestones later than their peers [90]. Collectively, these trends helped redefine the medical community’s thought of early infancy in relation to RTT. In response to Dr. Kerr’s qualitative study, the European Pediatric Society conference removed normal early infancy development as a necessary criterion for RTT diagnosis [1].

Despite its pros, retrospective parental analysis is by no means a flawless method. Most questionnaires and interviews take place a considerable time after the age of interest [93]. Parents participating in qualitative interviews or inquiry studies can also experience memory bias because they are now aware of their child’s disorder. Issues recalling or describing the origin of certain behaviors associated with RTT can affect the quantity and quality of information. With so much variability case by case, researchers might have issues extrapolating comparable data. Different home environments, proficiency in parental observation and reporting of a child’s behavior, and degrees of memory bias produce varied results. For example, the largest natural history study based on parental interviews portrays early RTT development differently from Dr. Kerr’s study. Of the 653 parents who participated in the study, 645 (99%) believed their daughters had experienced normal development despite their later diagnosis with classic RTT [95]. In this case, normal development was defined as acquiring fine motor and communication skills. This surprisingly high proportion may be due to retrospective memory bias or varied proficiency of parents observing their child’s behavior and development compared to a trained clinical researcher [1,95].

Diagnosis of RTT

RTT has well-established diagnostic criteria distinguishing classic and atypical RTT patients [1,96]. The four main criteria for RTT are partial or complete loss of purposeful hand skills, loss of previously acquired spoken language, gait abnormalities, and stereotypical hand motions. Loss of spoken language, in particular, is based on a patient’s best-acquired spoken language skill [97]. Classic RTT patients must not only meet all the main criteria and experience regression followed by recovery or stabilization but must also meet exclusion criteria. The exclusion criteria for classic RTT are brain injury secondary to trauma, severe infection linked to neurological issues, neurometabolic disease, or severe psychomotor abnormalities during the first six months of life. A classic RTT diagnosis must then be revisited if regression is not apparent by five years of age. In contrast, atypical RTT patients must meet at least two of the four main criteria, experience a regression period followed by stabilization or recovery, and fulfill 5 of the 11 supportive criteria for atypical RTT. Patients who present overlapping features with classic and atypical RTT but do not satisfy the criteria for either condition are classified as “Rett-like” in medical literature instead [98].

Despite the availability of diagnostic criteria for RTT, the median age at which a patient is confirmed to have RTT is still about three years old. Even the interquartile range of tentative RTT diagnosis is 2-4 years of age, which still lies outside the age range of normal development or stagnation in RTT's clinical progression. This relatively late diagnosis is the product of several factors. Foremost, RTT has overlapping clinical features with other medical conditions. These conditions include and are not limited to autism, cerebral palsy, hearing and vision problems, and phenylketonuria. Due to its prevalence of 1 in 10,000 females, RTT is too rare for physicians to diagnose without excluding these other conditions. In fact, RTT was only separated from autism spectrum disorder starting in the Diagnostic and Statistical Manual of Mental Disorders - Fourth Edition. RTT patients may also present features overlapping with other conditions during the later stages of RTT's progression. Development of autistic-like features of RTT patients during rapid regression and pseudo-stationary allow many to fit into the autism spectrum, for instance [56,66,99,100]. Besides its parallels to other medical conditions, RTT is absent from newborn screening currently. Assessing for MECP2 mutations as part of a newborn screen could be considered if a definite treatment for RTT becomes available. However, the only FDA-approved treatment for the disease is limited to RTT patients who are two years of age and older. RTT also lacks a cure currently. With the recognition of these diagnostic challenges and the need for improved treatment options, an emphasis has been placed on effective interventions for RTT.

Present perspectives on treatment of RTT

RTT lacks a cure, but treatments exist for the disorder’s symptoms and improving the quality of life of RTT patients [101]. Valproate, lamotrigine, levetiracetam, carbamazepine, and other anti-seizure medications are commonly prescribed for seizure relief. Serotonin reuptake inhibitors can treat altered behavioral patterns. A consistent sleep schedule and relaxing bedroom environment may address issues with falling asleep and abruptly waking during nighttime for patients with sleep disorders [101-103]. Communication interventions in educational, home, or rehabilitation settings are focused on improving choice-making, social communication, and communicative language [104-110]. In addition, physical therapy can encourage weight-bearing movement to promote osteoblastic activity and maintenance of vitamin D levels to protect the fragile bodies of RTT individuals that are four times more susceptible to bone fractures [76]. Physical therapy can incorporate assistive devices suited to the phenotype of a patient. Braces and casts can stabilize the hands and joints of patients with scoliosis, whereas walkers and wheelchairs can address more severe mobility limitations. However, one RTT treatment has now received FDA approval.

In 2023, trofinetide (Daybue) became the first FDA-approved treatment for RTT adult and pediatric patients who are two years of age and older. Trofinetide is a synthetic analog of glycine-proline-glutamate (GPE), a naturally occurring protein in the brain that has previously improved motor and respiratory function in MECP2 mice [111]. Its approval for human RTT patients is related to its ability to increase levels of insulin-like growth factor (IGF-1) and reduce inflammation in the brain. Multiple trials verify trofinetide’s safety, tolerability, and efficacy for RTT patients. A 42-day phase 2 trial featuring 82 patients who were ages 5 to 15 years old highlighted the safety and tolerability of different trofinetide dosages [112]. The highest trofinetide dosage administered (200 mg/kg twice daily) corresponded to statistically significant improvements in efficacy compared to a placebo and core RTT symptoms as measured by the Rett Syndrome Behavior Questionnaire (RSBQ), RTT-Clinician Domain Specific Concerns-Visual analog Scale (RTT-DSC-VAS), and Clinical Global Impression-Improvement (CGI-I) [112]. A phase 3 randomized clinical trial featuring female patients ranging from 5 to 20 years of age similarly supports the safety, tolerability, and tolerability of trofinetide [113,114]. Common treatment adverse events such as diarrhea (80.6% for trofinetide versus 19.1% for placebo), vomiting (27.0% for trofinetide versus 9.6% for placebo), and seizure (8.6% for trofinetide versus 5.3% for placebo) almost entirely ranged from mild to moderate severity [114]. In the same trial, trofinetide also exhibited statistically significant improvement in tested efficacy endpoints compared to a placebo. The trial’s coprimary efficacy endpoints were the changes from baseline to week 12 in the RSBQ and CGI-I scale scores. Moreover, its secondary efficacy endpoint was the change from baseline to week 12 in the Communication and Symbolic Behavior Scales Developmental Profile Infant-Toddler Checklist (CSBS-DP-IT) Social Composite score. The findings of this trial paired with FDA approval establish trofinetide as a treatment option at the forefront of addressing RTT’s pathophysiology, and other treatments may follow soon.

Future perspectives on treatment of RTT

In the near future, precision medicine could approach the level of current options for the treatment of RTT [55]. Precision medicine is an intervention approach based on the idea that not all RTT patients respond the same way to a specific treatment. Patient A could be identified as a better candidate for gene therapy compared to patient B, for example. This approach could produce a more personalized, effective treatment regimen for RTT by accounting for an RTT individual’s age, degree of XCI skewing, and specific mutation [55]. Gene therapy and X chromosome reactivation are thereby receiving significant attention. Table 1 provides an overview of these anticipated interventions that we will now discuss in more detail. 

**Table 1:** Overview of anticipated Rett syndrome interventions RTT: Rett syndrome; MECP2: methyl CpG binding protein 2

Treatment	Advantages	Limitations
Gene therapy	Gene therapy can reverse core RTT symptoms by introducing functional copies of MECP2 to cells exhibiting mutated MeCP2.	This intervention can be pathogenic in hypermorphic RTT patients. Specifically, it can result in MECP2 overexpression linked to disease phenotypes such as MECP2 duplication syndrome.
X chromosome reactivation	X chromosome reactivation can promote the re-expression of silenced MECP2 genes to increase levels of fully functional MeCP2 in cells.	This intervention can be pathogenic in the case of hypermorphic MECP2 mutations, and it impacts the expression of other X-linked genes.

Gene therapy is a precision medicine approach that has both capabilities and limitations. It can introduce new genetic material for genome editing or compensation for MECP2’s mutated protein. Previous mouse model studies support the potential applicability of MECP2-expressing lentiviral vectors used in gene therapy to human RTT patients. In one 2013 study, two MECP2-expressing vectors produced phenotypic improvements in MECP2-KO mice in spite of their different cell transduction rates [115]. A single-strand AAV9-MECP2 vector increased MECP2 expression in the brain when intracerebrally administered to neonatal MECP2-KO mice, improving its phenotype [115]. A scAAV9-pME (229 base pair)-MECP2 vector also improved the phenotype of MECP2-KO mice, specifically survival [115]. Another study featuring the MECP2-KO male and heterozygous female mice demonstrated that administration of scAAV9-pME (700 base pair)-MECP2 vector also corresponded to phenotypic improvements [116]. Nevertheless, caution about the current limitations of gene therapy still exists. Different MECP2-expressing vectors can exhibit variable transduction rates depending on their dosage and type, and low transduction rates limit phenotypic benefits [115,116]. Studies have also shown that MECP2 vector delivery can be pathogenic when healthy and mutated cells are transduced in females who have genetic mosaicism [117]. MECP2 overexpression is a cause of MECP2 duplication syndrome, another neurodevelopmental disorder [117].

In addition to gene therapy, reactivation of the silent X chromosome in female patients is another precision medicine approach that could approach current RTT treatments. MECP2 reactivation has already been achieved via different experimental means. The use of an X-inactive specific transcript (Xist) antisense oligonucleotide with a small molecule DNA methylation inhibitor can upregulate MECP2 in vitro, effectively restoring it [118]. MECP2 reactivation has also been observed in mouse cortical neurons that have received an intracerebral injection of activin A receptor type 1 (ACVR1) and pyruvate dehydrogenase kinase 1 (PDPK1) [119]. Additionally, Janus kinase/signal transducers and activators of transcription (JAK/STAT) pathway inhibitors have also demonstrated X chromosome reactivation capabilities in vitro. Despite these promising results, concerns about X chromosome reactivation exist [120]. One concern is the gene upregulation caused by X chromosome reactivation because MECP2 is not the only X-linked gene impacted. This aspect of X chromosome reaction has become a point of emphasis in recent investigations about the technique. For instance, Dr. Bhatnagar and his collaborators studied female Stanniocalcin 1 (STC1) -/- mice lacking random X chromosome inactivation and obtained promising results [121]. The STC1 -/- mice still experienced a normal life expectancy, and 98% of their X-linked genes were not overexpressed. Another concern is that X chromosome reactivation is pathogenic for certain MECP2 mutations. Increased MECP2 expression in female mice with hypermorphic mutations like R133C has been linked to MECP2 duplication syndrome, whereas T158 or R255X mice did not experience these adverse pathogenic effects when their MECP2 levels were elevated [122-124]. The effect of X-chromosome reactivation on different MECP2 mutations warrants further investigation consequently. These treatment developments show significant potential to improve the quality of life for patients and our understanding of RTT alongside previously discussed advancements in RTT research. Moreover, they demonstrate the degree to which our insight into RTT's pathophysiology has increased. Tables 2, 3 summarize significant studies that have advanced our understanding of RTT's underlying mechanisms thus far.

**Table 2:** Mouse studies discussed in this review RTT: Rett syndrome; MECP2: methyl CpG binding protein 2; JAK/STAT: Janus kinase/signal transducers and activators of transcription; FOXG1: forkhead box G1

References	Year Published	Key Findings
Asaka et al. [5]	2006	Decreased MeCP2 expression is linked to age-dependent changes in excitatory synaptic plasticity, and these alterations can be contributing factors to RTT.
Chao et al. [6]	2007	A lack of MeCP2 corresponds to reduced synaptic response in hippocampal glutamatergic neurons, while an increase of MeCP2 increased synaptic response. This trend demonstrates MeCP2 is integral to regulation of glutaminergic synapse formation.
Collins et al. [7]	2004	Strict regulation of MeCP2 levels exists in vivo because overexpression can be pathological as shown by mice with increased MeCP2 levels.
Moretti et al. [8]	2006	Hippocampus-dependent spatial memory, social memory, and contextual fear memory, and long-term plasticity may be inhibited in RTT mouse models. These effects suggest that MeCP2 is critical to learning and memory.
Nelson et al. [9]	2006	MeCP2 may be involved in the regulation of excitatory presynaptic function because it modulates gene expression.
Skene et al. [12]	2010	MeCP2 deficiency is linked to altered neuronal chromatin structure and histone H1 doubling, suggesting that MeCP2 may not be a gene-specific transcriptional repressor for neurons.
Chen et al. [13]	2015	Preferential dysregulation of genes exhibiting elevated mCH levels in the case of MeCP2 disorders suggests that MeCP2 binding to mCH loci is integral to neuronal gene expression. Moreover, MeCP2 has a higher affinity for mCH compared to Bdnf which is linked to RTT’s pathophysiology.
Cohen et al. [14]	2011	MeCP2 binds methylated CA sites located in long genes to repress gene expression. MECP2 mutations thereby may undermine expression of long genes in the brain.
Lagger et al. [16]	2017	The degree of MeCP2 binding to chromosomal DNA depends on density of mCAC and cytosine-guanine nucleotides (mCG). This outcome suggests that MeCP2 may incorporate mCAC and mCG to regulate gene transcription related to neuronal function.
Ebert et al. [17]	2013	Activity-dependent MeCP2 phosphorylation at T308 regulates NCoR complex and MeCP2 interactions. The absence of this phosphorylation may be one of the contributing factors to RTT.
Lewis et al. [18]	1992	A new protein has been identified that is separate from MeCP1 and can bind to DNA containing methyl-CpG pairs.
Kinde et al. [20]	2016	Disrupted methylation at CA sites is associated with decreased MeCP2 levels for genes that feature high methylated CA (mCA) concentration. Additionally, the degree of gene repression that Me CP2 engages depends on availability of methylated cytosine sites for it to bind to on a gene’s body.
Chahrour et al. [21]	2008	MeCP2 is involved in the modulation of gene expression in the hypothalamus and can act as a transcriptional activator and repressor.
Qiu et al. [25]	2012	MeCP2 plays a role in the regulation of activity-dependent synaptic scaling, and the dysregulation of this process may be linked to the pathophysiology of RTT.
Martynoga et al. [45]	2005	FOXG1 plays a key role in regulating precursor proliferation due to its regulation of fibroblast growth factor (FGF) signaling and differentiation.
Xuan et al. [46]	1995	BF-1 regulates neuroepithelial cell production and neuronal differentiation timing. It thereby may be involved in regulation of the telencephalon’s morphogenesis.
Vezzali et al. [47]	2016	IGF-1 signaling regulates activation of Cdkn1a transcription by FOXO1. Additionally, FOXG1 is also linked to the activation of the transcription of Kcnh3, a gene that may be involved in pathology of FOXG1 syndrome.
Chen et al. [65]	2001	MeCP2 plays a critical role in mature neurons because dysfunction of these neurons and onset of RTT-like symptoms are observed in the case of MeCP2 deficiency.
Guy et al. [67]	2001	The overlap of delayed symptom onset in RTT patients and RTT mouse models suggests that the MeCP2 deficiency may disrupt the stability of brain function.
Bedogni et al. [69]	2016	MeCP2 regulates transcriptional mechanisms governing development of the cerebral cortex. Neurological phenotypes associated with MeCP2 deficiency could be the product of adverse events that occur even before pathology is apparent.
Weng et al. [71]	2011	Progressive functional synaptic impairment is a major feature of RTT brains, and the possibility of plasticity restoration as shown by memantine administration partially restoring short-term plasticity.
Lozovaya et al. [73]	2019	Treatment with bumetanide can mitigate alterations observed at birth. This discovery supports the idea that birth should be a considered a critical period in RTT.
Calfa et al. [75]	2015	Loss-of-function MECP2 mutations can promote hyperactivation of the hippocampal network by disrupting excitation/inhibition balance onto CA3 pyramidal neurons.
Dani et al. [78]	2005	A shift in cortical excitation and inhibition balance is linked to reduced spontaneous activity of pyramidal neurons in Mecp2-mutant mice. MECP2-mutant cortices may exhibit decreased miniature excitatory postsynaptic current (mEPSC) amplitudes.
Wood et al. [79]	2009	MeCP2 deficiency in postsynaptic cortical pyramidal neurons can promote synaptic pathological alterations in excitatory intracortical circuits.
Abdala et al. [80]	2008	Increasing GABA transmission decreases respiratory arrhythmia in RTT mouse models, whereas synaptic inhibition within the Kölliker-Fuse area (KF) can increase respiratory arrhythmia.
Durand et al. [82]	2012	Sensory deprivation and deletion of NR2A can rescue inhibitory hyperconnectivity and cortical function. This phenomenon suggests that vision can act as a sensitive biomarker for progressive cortical dysfunction, making it relevant for circuit-based therapies for MeCP2 deficiency.
Mierau et al. [83]	2016	The timeline of N-methyl-D-aspartate receptor (NMDAR) is specific to cell-type, and MeCP2 plays a cell-type specific role in the composition of NMDAR subunits.
Tropea et al. [111]	2009	Insulin-like Growth Factor 1 (IGF-1) can lead to partial recovery of synaptic amplitude and spine density, stabilization of cortical plasticity, and increase in PSD-95 levels. These outcomes suggest that IGF-1 could be used in a pharmacologic RTT intervention.
Gadalla et al. [115]	2013	Intravenous injection of a self-complementary adeno-associated virus (AAV) vector into juvenile mice resulted in improved survival, supporting the practicality of gene therapy.
Garg et al. [116]	2013	AAV9 that is self-complementary can stabilize and even alleviate symptoms found in female RTT mouse models. This outcome suggests a potential gene therapy option for RTT female patients.
Carrette et al. [118]	2018	Mixed-modality approach that combined small molecule inhibitor of DNA methylation and Xist antisense oligonucleotide can upregulate MECP2. These results support the feasibility of a mixed-modality treatment approach for X-linked disorders.
Przanowski et al. [119]	2018	Intracerebroventricular injection of XCI factors (XCIFs) can reactivate MECP2 in cerebral cortical neurons of adult mice, supporting the practicality of pharmacological reactivation of MECP2.
Lee et al. [120]	2020	A pathway that could potentially be targeted to help restore MECP2 gene expression is the JAK/STAT pathway. Its inhibition could thereby be incorporated into RTT treatment.
Bhatnagar et al. [121]	2014	Mechanism(s) exist that regulate X-linked gene expression by compensating for XCI deficiency.
Vermudez et al. [122]	2022	Introduction of a wild-type MECP2 transgene into a RTT mouse model with a hypermorphic R133C mutation not only alleviates core RTT symptoms but also has adverse effects due to MeCP2 overexpression.
Lamonica et al. [123]	2017	Increasing MeCP2 T158M protein expression levels can alleviate RTT-like phenotypes. For this reason, therapies that target MeCP2 T158 expression could be a viable treatment option.
Pitcher et al. [124]	2015	The R255X mutation of MECP2 is responsive to nonsense suppression therapy, and rescue is thereby possible to an extent when the MeCP2 is re-expressed.

**Table 3:** Human studies discussed in this review RTT: Rett syndrome; MECP2: methyl CpG binding protein 2; FOXG1: forkhead box G1

Reference	Year Published	Number of Subjects	Key Findings
Neul et al. [3]	2008	245	Specific MECP2 mutations correspond to different clinical severities of RTT.
Bahi-Buisson et al. [11]	2010	205	Patients with FOXG1 mutations exhibit features that are compatible with the congenital variant of RTT, including severe encephalopathy.
Armstrong et al. [28]	2001	1	MECP2 mutation R270X was identified in a male patient exhibiting somatic mosaicism.
Shah et al. [29]	2021	1	RTT-linked pathogenic variant c.538C > T (p.R180*) was identified in males who exhibited post-zygotic de novo somatic mosaicism.
Takeguchi et al. [30]	2020	1	Nonsense and mosaic variant was found in MECP2 exon 1 of male patient, who exhibited less severe RTT symptoms than male patients with mosaic variants in MECP2 exon 3 or 4.
Tokaji et al. [31]	2018	1	Duplication in MECP2 exon 1 that results in premature stop codon has been identified in a male patient.
Topcu et al. [32]	2002	1	RTT-linked MECP2 mutation can produce a similar phenotype in males due to somatic mosaicism.
Burd et al. [33]	1991	297	From participants, only females satisfied the required criteria to be diagnosed with RTT.
Elia et al. [41]	2008	8	CDKL5 gene mutations may correspond to severe mental retardation and early-onset seizures. These symptoms can potentially be used in screening for CDKL5 mutations.
Tarquinio et al. [42]	2017	1205	Longitudinal analysis suggests lifetime risk of epilepsy for RTT patients is higher than previously reported in medical literature.
Fehr et al. [43]	2013	1006	Most analyzed CDKL5 mutation cases did not meet criteria for atypical RTT diagnosis, suggesting that CDKL5 should be defined as a separate condition from RTT.
Scala et al. [44]	2005	2	CDKL5 mutations are linked to a RTT variant characterized by early convulsion development.
Mitter et al. [51]	2018	83	Variability in the severity of psychomotor and neurological phenotypes exists among FOXG1 variants.
Ariani et al. [53]	2008	2	FOXG1 mutations can exist in patients afflicted with congenital variant of RTT. FOXG1 and MeCP2 also may have overlapping mechanisms during neuronal development.
Anderson et al. [57]	2014	396	A detailed estimation of survival rates based on follow-up data has now been created, and these estimations suggest multidisciplinary services can improve health and wellbeing of patients.
Glaze et al. [58]	2010	602	The onset and occurrence of seizure is age-related and influenced by the mutation type of patient.
Nissenkorn et al. [59]	2015	1248	MeCP2 may exhibit a site-specific effect on epileptic pathway because different MECP2 mutations impact epilepsy differently.
Cardoza et al. [64]	2011	89	Discrepancy in the classification of epileptic seizure classifications can exist among clinicians, suggesting limitations in only using clinical history to classify epileptic seizure type in RTT patients.
Carter et al. [68]	2008	23	RTT brains exhibit volume reductions that can impact parietal lobe gray matter, cortical white matter, and anterior front lobe volumes.
Bernardo et al. [72]	2020	34	RTT patients exhibit reduced short-interval intracortical inhibition, increased intracortical facilitation, reduced long-interval intracortical inhibition, and disrupted long-term potentiation (LTP) plasticity.
Jian et al. [74]	2007	162	Seizure frequency is age dependent for RTT cases. It also influenced by the mutation type of an individual and the presence of severe early developmental issues.
Marschik et al. [85]	2009	1	Development of hand stereotypies, asymmetrical eye opening, abnormal facial expressions, and abnormal movement suggest that preserved speech variant of RTT can appear during first few months of life.
Pokorny et al. [86]	2016	8	A collection of acoustic features was identified to distinguish altered development linked to RTT from typical development.
Pokorny et al. [87]	2018	1	More than half of the linguistic vocalizations from video recordings of a RTT patient as an infant were considered ‘atypical’ due to their prosodic, voice, and spectral quality.
Marschik et al. [88]	2014	1	An individual with preserved speech variant (PSV) of RTT exhibited an altered developmental speech-language trajectory despite achieving speech-language milestones.
Downs et al. [89]	2008	99	Individuals with p.R133C, p.R255X, or p.R294X mutations seem to exhibit less impaired motor skills compared to individuals with a large deletion mutation or p.R270x mutation.
Marschik et al. [90]	2014	3	The control toddler that did not have RTT exhibited more communicative gestures and linguistic vocalizations than toddlers diagnosed with forms of RTT.
Percy et al. [95]	2010	819	All classic RTT study participants fulfilled main criteria, whereas variant RTT participants satisfied at least two of four main criteria and 5 of the 11 supportive criteria in the revised diagnostic criteria for RTT. These outcome highlights the validity of this diagnostic criteria.
Schonewolf-Greulich et al. [98]	2019	35	Rett-like phenotypes have diagnostic overlaps with other conditions and clinical and genetic heterogeneity. Moreover, the first pathogenic KCNB1 variant connected to Rett-like phenotype has been identified.
Vignoli et al. [101]	2012	130	Older patients that exhibited C-terminal deletions in addition to R294X or R133C mutations demonstrated less clinical severity of RTT.
Sadeghi, Shevell [103]	2018	3	There is a necessity for established guidelines for genetic testing that considers patients’ presentations instead of a specific element of their histories.
Fabio et al. [104]	2016	34	Long-term training (LTT) can improve the behavioral parameters of RTT patients. It can also positively impact the brain by increasing beta activity, decreasing activity, and reestablishing leftward symmetry.
Hetzroni et al. [105]	2002	3	Individualized multimedia programs for RTT patients can result in partial knowledge retention and a steady learning curve.
Koppenhaver et al. [106]	2001	6	Storybooks can encourage RTT patients to be active in communication interactions and engage in a larger range of communication modes.
Skotko et al. [107]	2004	4	Parent-child storybook interactions can be used to teach RTT patients to communicated in a more meaningful manner.
Fabio et al. [108]	2009	20	When presented with the task of discriminating previously presented stimuli from distractors, female RTT patients learned at a quicker rate when their stereotypies were contained.
Fabio et al. [109]	2018	3	Transcranial direct current stimulation (tDCS) and cognitive empowerment can enhance language abilities, including speech production and comprehension, for RTT patients.
Ryan et al. [110]	2004	3	Multi-modal cueing can facilitate communicative responses of children diagnosed with RTT. However, more frequent communicative interactions do not necessarily result in increased responses from these individuals.
Glaze et al. [112]	2019	82	Different dose levels of trofinetide can be well tolerated and safe. Moreover, trofinetide can correspond to statistically significant improvement in core RTT symptoms compared to placebo, supporting need for further trials.
Neul et al. [114]	2023	187	Significant improvement in efficacy endpoints was observed in patients treated with trofinetide compared to placebo, suggesting trofinetide’s effectively treats core symptoms of RTT.
Smeets et al. [117]	2005	107	Dystonia is linked to severe spine deformation and apparent from childhood in RTT females with C-terminus hot spot deletions.

## Conclusions

RTT, the most common cause of severe intellectual disability in females, still has many unresolved questions. Despite these unknowns, including the lack of a cure and methods for earlier diagnosis, research efforts in the field have not been dampened. Increased understanding of the pathophysiology of RTT, especially MECP2’s link and involvement in an ever-increasing range of cell types and physiological processes, has encouraged novel investigations of the disease. Video recording studies, retrospective parental analysis, and MECP2 mutant mouse model studies are just a few of the investigative techniques that derive most of their value from an improved grasp of the effects of MeCP2 deficiency in the case of loss-of-function MECP2 mutations. An improved grasp of MECP2’s properties has and continues to influence treatment developments, including precision medicine and the FDA-approved trofinetide, aimed at improving the quality of life of RTT patients. Collectively, MECP2 and the methods and observations it has inspired are inspiring our hopes of fully grasping RTT.
